# Working memory deficits in patients with idiopathic restless legs syndrome are associated with abnormal theta‐band neural synchrony

**DOI:** 10.1111/jsr.13287

**Published:** 2021-02-09

**Authors:** Kwang Su Cha, Jun‐Sang Sunwoo, Jung‐Ick Byun, Tae‐Joon Kim, Jung‐Won Shin, Kyung Hwan Kim, Ki‐Young Jung

**Affiliations:** ^1^ Department of Neurology Seoul National University Hospital Seoul South Korea; ^2^ Department of Neurosurgery Seoul National University Hospital Seoul South Korea; ^3^ Department of Neurology Kyung Hee University Hospital at Gangdong Seoul South Korea; ^4^ Department of Neurology Ajou University School of Medicine Suwon South Korea; ^5^ Department of Neurology CHA Bundang Medical Center CHA University Seongnam South Korea; ^6^ Department of Biomedical Engineering College of Health Science Yonsei University Wonju South Korea; ^7^ Neuroscience Research Institute Seoul National University College of Medicine Seoul South Korea; ^8^ Sensory Organ Research Institute Seoul National University Medical Research Center Seoul South Korea

**Keywords:** cognitive deficits, event‐related potentials, restless legs syndrome, theta‐band activity, theta‐band phase synchrony, working memory

## Abstract

Cognitive impairment, particularly prefrontal function, has been reported in patients with restless legs syndrome. However, working memory performance in patients with restless legs syndrome remains uncertain. The present study aimed to examine working memory performance in patients with restless legs syndrome by investigating electroencephalography theta‐band oscillations within task‐relevant brain regions and the synchronization among oscillations during a working memory task. Twelve female idiopathic patients with restless legs syndrome and 12 female healthy controls participated in this study. Nineteen‐channel electroencephalography data were recorded while participants performed a Sternberg working memory task. We analysed event‐related theta‐band activity and interregional theta‐band phase synchrony during the memory retrieval phase. The spatial pattern of theta‐band phase synchrony was quantified using graph theory measures, including the clustering coefficient, characteristic path length, and small‐world propensity. Considerable increases in theta‐band activity and theta‐band phase synchrony were observed at 600–700 ms in controls and at 650–750 ms in restless legs syndrome subjects after the probe item was presented. During this period, induced theta‐band activity showed lower with borderline significance in the restless legs syndrome subjects than in the controls regardless of channel location (*F*_4,88_ = 3.92, *p* = .06). Theta‐band phase synchrony between the frontal and posterior regions was significantly reduced in the restless legs syndrome subjects. Inefficiency in both global and local networks in the restless legs syndrome subjects was revealed by the decreased small‐world propensity (*t*_22_ = 2.26, *p* = .03). Small‐world propensity was negatively correlated with restless legs syndrome severity (*r *= −.65, *p* = .02). Our findings suggest that patients with restless legs syndrome have multiple deficits in cognitive processes, including attentional allocation, evaluation of incoming stimuli, and memory manipulation of encoded information during a working memory task. Abnormal local theta‐band neural synchrony and global theta‐band neural synchrony may underlie the neurophysiological mechanism of the working memory dysfunction associated with restless legs syndrome.

## 1 INTRODUCTION

Restless legs syndrome (RLS) is a sensorimotor neurological disorder that is characterized by an irresistible urge to move the legs during rest mostly in the evening, usually accompanied by sensory discomfort (Allen & Earley, 2001). In addition to sensorimotor symptoms and sleep disturbance, cognitive impairment, particularly prefrontal function, has been reported in patients with RLS (Jung, 2015; Pearson et al., 2006). Working memory (WM) provides temporary storage and the ability to manipulate the information necessary for complex cognitive tasks (Baddeley, 1992). The frontal cortex plays an important role in executing WM processes (Smith & Jonides, 1999). Thus, it can be inferred that patients with RLS may have a deficit in WM function. However, the majority of neuropsychological studies, except one (Galbiati et al., 2015), have reported that patients with RLS have no deficits in WM function (Celle et al., 2010; Fulda et al., 2010; Moon et al., 2014; Zhang et al., 2017).

An event‐related potential (ERP) study suggested WM dysfunction in RLS by demonstrating abnormal neural activities in patients with RLS while they performed a Sternberg WM task. Patients with RLS exhibited lower P3 amplitudes at parietal regions than controls during the memory retrieval phase. The P3 amplitude was negatively correlated with the duration of RLS illness, reflecting cortical dysfunction in patients with RLS due to repeated RLS symptom attacks (Kim et al., 2014). The discrepancy between the neuropsychological and neurophysiological test results may be because ERPs are a more sensitive measure for objectively assessing specific cognitive function. Thus, an ERP study may contribute to the characterization of the detailed pathophysiological mechanism of WM dysfunction associated with RLS.

In addition to conventional ERP analysis in the time domain, neural oscillations within the task‐relevant cortical regions and synchronization among these oscillations provide useful information for investigating the dynamic changes in synchronized neural activities and the network underlying cortical information processing (Vaz et al., 2019). Neural oscillations in the theta band have been implicated in various aspects of WM processing, including encoding, maintenance and retrieval (Jensen & Tesche, 2002; Ko et al., 2012; White et al., 2013).

Rhythmic theta‐band activity (TBA) is the dominant rhythm in memory processing. Hippocampal theta‐band oscillations during memory retrieval, in particular, are crucial for reactivation of encoded memory traces (Itthipuripat et al., 2013; Klimesch, 1999). Furthermore, long‐range neural synchrony between distant brain regions plays a key role in planning multistep actions for memory processing (Ishino et al., 2017; Sauseng et al., 2010; Watrous et al., 2013). Encoded memory traces should be reactivated by hippocampal and medial prefrontal cortex (mPFC) theta‐band oscillations for successful memory retrieval (Backus et al., 2016). Consequently, characterization of the temporal fluctuations in theta‐band neural oscillations in multiple cortical regions may help to reveal the details of memory retrieval. The patterns of regional neural synchrony and interregional neural synchrony in the theta band can be investigated by analysing event‐related spectral perturbation (ERSP) and phase synchrony between electroencephalogram (EEG) signals, and may lead to a more complete understanding of WM processing.

We hypothesized that TBA and interregional theta‐band phase synchrony (TBPS) may be significantly altered during a WM task in patients with RLS compared with the activity and TBPS in healthy controls. To address this hypothesis, we evaluated the spatiotemporal characteristics of local neural synchrony and global neural synchrony in the theta band by analysing TBA and TBPS while participants performed a Sternberg WM task. Graph theory analysis was also performed to characterize the spatial patterns of abnormal functional connectivity in patients with RLS.

## 2 METHODS

### 2.1 Subjects

Twelve drug‐naïve female patients with idiopathic RLS and 12 age‐matched female healthy controls participated in this study. All subjects completed structured sleep questionnaires that included the Insomnia Severity Index (ISI; Bastien et al., 2001), the Epworth Sleepiness Scale (ESS; Johns, 1991), the Beck Depression Inventory‐II (BDI‐II; Sung et al., 2008), and the Pittsburgh Sleep Quality Index (PSQI; Buysse et al., 1989). Patients with RLS were diagnosed based on the diagnostic criteria established by the International RLS Study Group (IRLSSG; Allen et al., 2003), and assessed in person by a neurologist with the validated Korean‐language version of the John Hopkins Telephone diagnostic questionnaire (Cho et al., 2007). RLS mimics and other comorbidities were carefully excluded. All participants had no prior treatment for RLS. Detailed inclusion and exclusion criteria for patients with RLS and healthy controls were described in our previous study (Kim et al., 2014). Each subject provided written informed consent prior to participation in this protocol. The Institutional Review Board of Seoul National University Hospital approved all procedures (IRB no. 1705–118–855).

### 2.2 Sternberg WM paradigm

**FIGURE 1:**
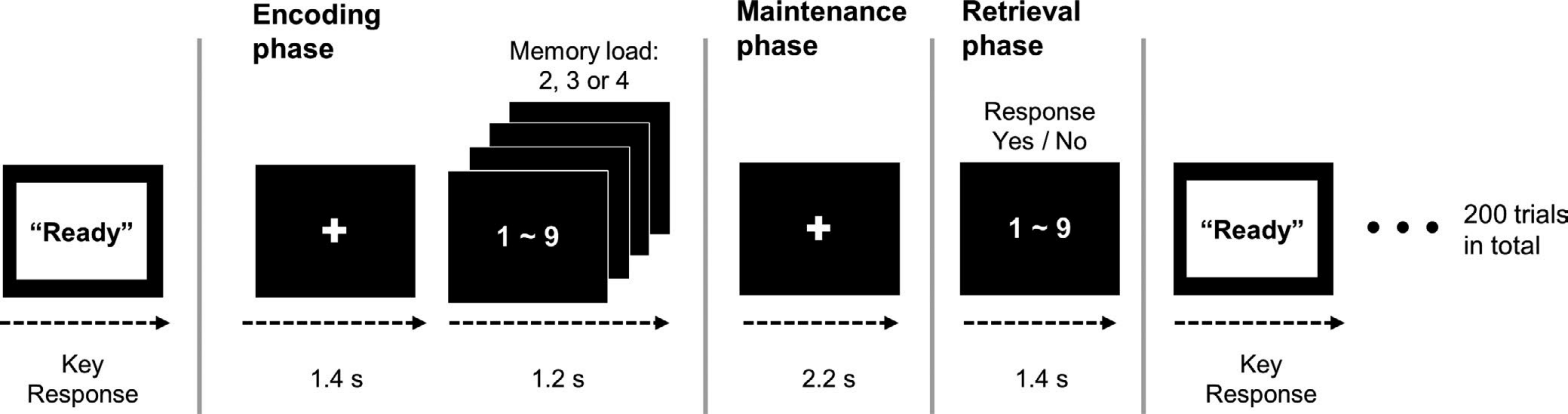
Schematic illustration of the Sternberg working memory (WM) task. After presenting a visual orienting cue sign, subjects were requested to monitor a series of digit numbers. The stimuli consisted of white numbers (from 1 to 9) presented on a black background and were sequentially presented for 1.2 s with a black screen presented for 0.2 s between each number according to the number of memory sets (2, 3 or 4). After a 2‐s maintenance phase (black screen), a probe stimulus was shown for 2 s, and subjects were required to press a button corresponding to whether the probe stimulus was included in the numbers in the memory sets. Subjects responded with either their left (matched items) or right hand (unmatched items). The experiment consisted of a total of 200 trials. Memory sets were randomly presented

Subjects performed a modified Sternberg WM task (Sternberg, 1966). After presenting a visual orienting cue sign, a series of digits was presented on a screen (Figure 1). The stimuli consisted of white numbers (from 1 to 9) presented on a black background, and were sequentially presented for 1.2 s with a black screen shown for 0.2 s between the presentation of the numbers. During the encoding phase, either two, three or four stimuli were presented according to the level of memory load. After a 2‐s maintenance phase (black screen), a probe stimulus was shown for 2 s, and subjects were required to press a button corresponding to whether the probe stimulus was included in the numbers in the memory sets that were presented previously in the encoding phase. The subjects were instructed to respond with either their left (matched items) or right hand (unmatched items). Accuracy of task performance (hit rates, HRs) and reaction times (RTs) of correct responses were measured for the behavioural response. The experiment consisted of a total of 200 trials. Memory sets were randomly presented.

### 2.3 EEG recording

Electroencephalogram signals were recorded using 19 electrodes over the entire scalp according to the international 10–20 system. Linked mastoid electrodes were used as a reference. Electrooculogram (EOG) activity was recorded via a bipolar derivation consisting of two electrodes on the left and right outer canthi to obtain a reference for ocular artefact removal. The impedances of all electrodes were reduced to below 10 kΩ. The EEG signals were amplified and filtered by a bandpass filter with cut‐off frequencies of 0.1–70 Hz, and then stored at a sampling rate of 400 samples per s. The visual stimuli were presented on a 17‐inch LCD monitor using commercial software (PRESENTATION; Neurobehavioral Systems, Berkeley, CA, USA). The distance between the subjects’ eyes and the monitor was 75 cm, and the visual angle was 1.91°.

### 2.4 EEG preprocessing

The EEG data were downsampled to 200 Hz to reduce the computational burden. The EEG waveforms were segmented from −200 ms to 1,500 ms based on the probe stimulus during the retrieval phase. Single‐trial waveforms that were severely contaminated by non‐stereotyped artefacts, such as drift or high‐frequency noise, were removed by visual inspection. Independent component analysis was performed to correct stereotyped artefacts such as ocular and muscular artefacts (Jung et al., 2000). Additionally, single‐trial waveforms were excluded from further analysis if the absolute value of the EOG exceeded 100 µV. The remaining waveforms were rereferenced against an averaged reference. We excluded all the error trials from analysis.

### 2.5 Event‐related spectral perturbation

To identify temporal changes in the spectral characteristics of ERPs, we performed time−frequency analysis based on continuous wavelet transform (CWT) with the Morlet wavelet coefficient as a mother wavelet function (Tallon‐Baudry et al., 1996). The number of cycles in the CWT linearly increased based on the frequency of interest, and ranged from 4 at the lowest frequency (1 Hz) to 13.5 at the highest frequency (100 Hz). The ERSP patterns of single‐trial ERPs in the theta band were obtained by averaging the time−frequency distribution of wavelet coefficient magnitudes over 19 electrodes. Then, the ERSP patterns were transformed to the relative ratio of power change with respect to the baseline interval. The evoked TBA was obtained from the ERSP pattern of the grand‐averaged ERP, and the induced TBA was calculated by subtracting the evoked TBA from the average of single‐trial TBAs. The temporal and frequency ranges of induced TBA were defined as 600–750 ms and 4–6 Hz, respectively.

### 2.6 Weighted phase lag index

For the functional connectivity analysis, long‐range phase synchronizations between EEG signals were calculated using the weighted phase lag index (wPLI), which is based on the imaginary component of the cross‐spectrum between a pair of EEG signals (Vinck et al., 2011), and is known to be minimally affected by volume conduction. To extract the instantaneous phase perturbation of the theta‐band oscillations (4–6 Hz) within EEG signals, a short‐time Fourier transform with a 512‐point fast Fourier transform, a 100‐point Hanning window and a 99‐point overlap was adopted. The cross‐spectrum between EEGs from electrodes *i* and *j*, *X_i,j_*, was calculated using the extracted complex‐valued Fourier spectra vector *Z* as follows: $X_{i,j}=Z_{i}Z_{j}^{*}$.

Then, the wPLI between the two electrodes was calculated using Equation (1):

(1) $$wPLI_{i,j}=\frac{\left|E\left\{\left|I\left\{X_{i,j}\right\}\right|\text{sgn}\left(I\left\{X_{i,j}\right\}\right)\right\}\right|}{E\left\{\left|I\left\{X_{i,j}\right\}\right|\right\}}$$

$I\left\{X_{i,j}\right\}$ is the imaginary part of the cross‐spectrum *X* between signals *i* and *j*. sgn(∙) denotes the sign function. Here, we selected 15 representative electrodes distributed across the scalp (Fp1, Fp2, F3, Fz, F4, C3, Cz, C4, T7, T8, P3, Pz, P4, O1 and O2) for the wPLI calculation. Four lateral electrodes (F7, F8, P7 and P8) were excluded from the analysis due to concerns that the wPLI could be disturbed by artefacts. The range of the wPLI is between 0 and 1, which indicates no coupling and perfect phase locking between the pair of EEG signals, respectively.

### 2.7 Graph theory analysis

We calculated graph theory measures, including the weighted clustering coefficient (*C*), the characteristic path length (*L*) and the network small‐world propensity (SWP) to analyse the network characteristics of the spatial pattern of interregional phase synchrony (Rubinov & Sporns, 2010; Stam et al., 2009). The nodes of the graph consisted of 19 electrodes. The edges of the graph were determined by all electrode pairs included in the functional connectivity analysis. wPLIs between every possible electrode pair were used for analysis.

The weighted *C* is used as an index of local connectivity, as it quantifies the intensities of the subgraphs of a node (Onnela et al., 2005). The weighted *C* at node *i* is defined by Equation (2) as follows:

(2) $$C=\frac{1}{N}\sum_{i=1}^{N}{\tilde{C}}_{i},{\tilde{C}}_{i}=\frac{2}{k_{i}\left(k_{i}‐1\right)}\sum_{j,k}{\left(\omega_{i,j}\omega_{j,k}\omega_{k,i}\right)}^{1/3}$$

where ω*_i,j_* indicates the adjacency between two nodes *i* and *j*. *N* is the total number of nodes in the network. $k_{i}$ is the degree of node *i*. An increase in the weighted *C* indicates higher local efficiency.

The weighted *L* quantifies the average of the shortest distances from one node to all other nodes in the network (Rubinov & Sporns, 2010). The weighted shortest distance $d_{i,j}$ is defined as the smallest inverse of the sum of wPLIs of connecting edges between *i* and *j*. A global measure of the functional interaction of the network *L* is defined as Equation (3):

(3) $$L=\frac{1}{N\left(N‐1\right)}\sum_{i,j\in N,i\neq j}\frac{1}{d_{i,j}}$$

with the total number of a node *N* in the network. A decrease in weighted *L* denotes higher global efficiency.

Small‐world propensity indicates a small‐world‐like network architecture, which is characterized by a weighted network with high *C* and small *L*. SWP was computed by the ratio of $C_{\text{norm}}$ and $L_{\text{norm}}$ Equation (4) (Humphries & Gurney, 2008):

(4) $$\text{SWP}=\frac{C_{\text{norm}}}{L_{\text{norm}}}=\frac{C/C_{\text{rand}}}{L/L_{\text{rand}}}$$

where $C_{\text{rand}}$ and $L_{\text{rand}}$ were generated by averaging the edge weights of 50 random networks, which were constructed by randomly reshuffling the edge pattern of an original network. A high SWP indicates that the functional connectivity network is efficient for interregional communication.

### 2.8 Statistical analysis

Two‐way repeated measures analysis of variance (ANOVA) was used for the statistical analysis of the induced TBA within the subregion of the time−frequency space determined above (600–750 ms, 4–6 Hz). The electrodes were grouped into three regions of interest (ROIs), i.e. frontal (F3, Fz and F4), central (C3, Cz and C4) and parietal (P3, Pz and P4) regions. The within‐subject variables were the ROI (frontal, central and parietal) and memory load size (2, 3 and 4). The between‐subject variable was the subject group (RLS and control).

For the comparison of functional connectivity (wPLI) patterns between groups, network‐based statistics (NBS) were employed (Zalesky et al., 2010). This method resolves the multiple comparisons problem for the cortical connectome by controlling the familywise error rate at the subnetwork level rather than at the level of individual connections. The initial univariate threshold (*t* = 3) for group comparisons was adopted to binarize the statistical matrix of all connections. Data surrogation was repeated 1,000 times to obtain a null distribution. Finally, the observed size of the component corresponded to *p* = .010.

Independent sample *t*‐tests were performed to compare the graph theory measures (C, *L* and SWP). Relationships between SWP, TBA and clinical variables, including age, disease duration, IRLS score, ISI score, PSQI score and BDI score, were investigated using Spearman's correlation coefficients.

## 3 RESULTS

### 3.1 Clinical and sleep‐related variables

Age, sex and body mass index were matched between the RLS group and the control group (Table 1). The IRLSSG rating scale (IRLS) score in the RLS group (30.75 ± 4.77) indicated severe RLS symptomatology. The BDI‐II scores were significantly higher in the RLS group than in the control group (*t*_22 _= −2.64, *p* = .02, *d* = −1.08). The ISI and PSQI scores were significantly higher in the RLS group (*t*_22 _= −6.79, *p* < .01, *d* = −2.77; *t*_22 _= −5.24, *p* < .01, *d* = −2.14, respectively), indicating poor sleep quality.

**TABLE 1:** Patient demographic data and sleep questionnaire results

	Control	RLS	*t*‐value	*p*‐value	Cohen’s *d*
Clinical characteristics					
Age (years)	49.25 (7.34)	53.42 (8.77)	−1.262	.220^a^	−0.516
Education (years)	13.17 (2.72)	11.33 (2.42)	1.741	.096	0.715
BMI (kg m^−2^)	22.08 (2.38)	22.03 (2.38)	0.051	.959	0.021
Ferritin (mcg L^−1^)	−	131.23 (66.83)	−	−	
Sleep‐related questionnaires					
IRLS	−	30.75 (4.77)	−	−	
ESS	5.08 (1.93)	5.83 (2.86)	−0.754	.459	−0.307
ISI	2.92 (2.15)	18.00 (7.39)	−6.792	.001	−2.771
PSQI	4.17 (2.04)	14.33 (6.40)	−5.243	.001	−2.139
BDI‐II	7.75 (5.99)	16.50 (9.77)	−2.644	.015	−1.080

Data are shown as mean (standard deviation).

BDI‐II, Beck Depression Inventory‐II; BMI, body mass index; ESS, Epworth Sleepiness Scale; IRLS, International RLS Severity Scale; ISI, Insomnia Severity Index; PSQI, Pittsburgh Sleep Quality Index; RLS, restless legs syndrome.

^a^ Independent samples *t*‐test.

### 3.2 Behavioural results

Table 2 shows the behavioural results of the WM task. The mean HR for all memory load sizes was 96.43 (± 2.10)% in the control group, and 96.35 (± 1.70)% in the RLS group (*t*_22_ = 0.11, *p* = .91, *d* = 0.04). The mean RT was 671.33 (± 60.46) ms in the control group and 846.81 (± 186.10) ms in the RLS group (*t*_22 _= −3.11, *p* < .01, *d* = −1.26). The patients with RLS showed significantly prolonged RT in all memory loads compared with controls (load 1: *t*_22 _= −2.58, *p* = .02, *d* = −1.05; load 2: *t*_22 _= −3.25, *p* < .01, *d* = −1.33; load 3: *t*_22 _= −3.27, *p* < .01, *d* = −1.34). However, there were no differences in HR for any memory load between groups (load 1: *t*_22 _= −0.61, *p* = .55, *d *= −0.25; load 2: *t*_22_ = 0.80, *p* = .43, *d* = 0.33; load 3: *t*_22 _= −0.03, *p* = .98, *d* = −0.01).

**TABLE 2:** Behavioural results during memory retrieval phase

	Control	RLS	*t*‐value	*p*‐value	Cohen’s *d*
HR (%)					
Load 2	96.81 (2.25)	97.34 (1.96)	−0.608	.550^a^	−0.251
Load 3	96.96 (2.42)	96.14 (2.62)	0.800	.432	0.325
Load 4	95.53 (3.66)	95.57 (3.61)	−0.026	.980	−0.011
RT (ms)					
Load 2	630.32 (63.89)	789.15 (203.32)	−2.582	.023	−1.054
Load 3	673.05 (60.58)	844.64 (172.56)	−3.250	.006	−1.327
Load 4	710.62 (72.92)	906.65 (194.22)	−3.273	.006	−1.336

Data are shown as a mean (standard deviation).

HR, hit rate; RLS, restless legs syndrome; RT, reaction time.

^a^ Independent samples *t*‐test.

### 3.3 Theta‐band activity

**FIGURE 2:**
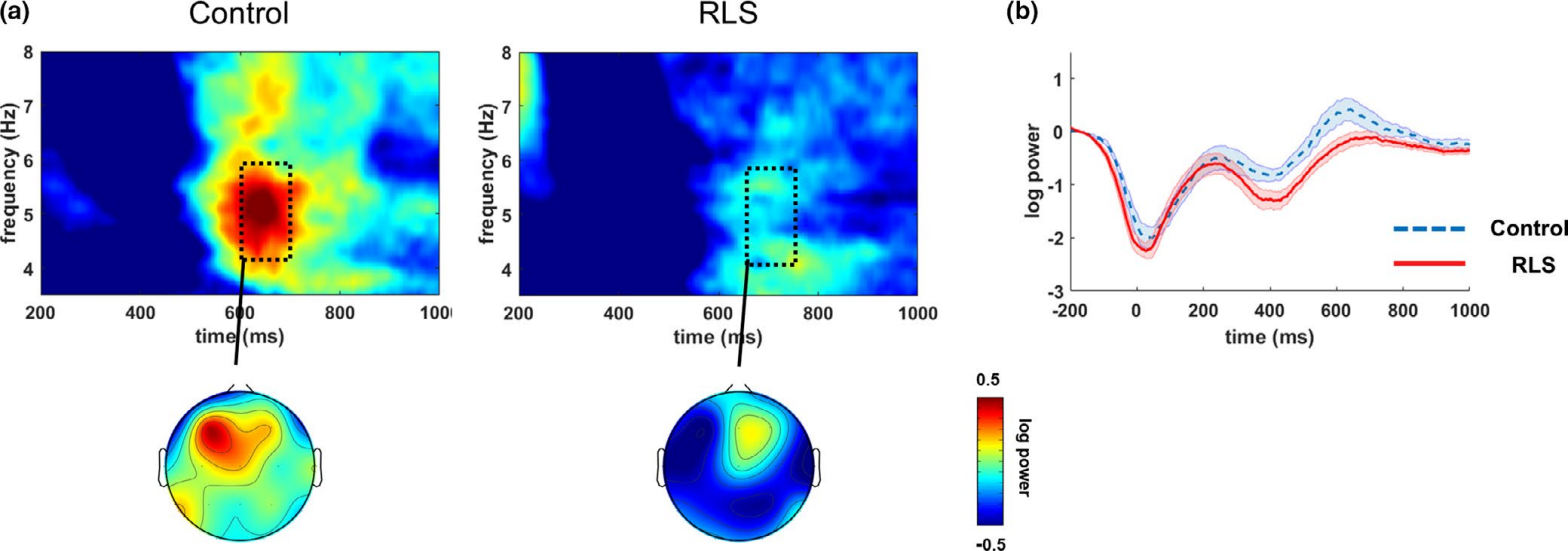
Induced theta‐band activity (TBA) during the memory retrieval phase. (a) Time−frequency representations of induced TBA in the frontal region (F3, Fz and F4). The topographical distribution shows induced TBA at 600–700 ms in control subjects and at 650–750 ms in patients with restless legs syndrome (RLS) after the probe item was presented. (b) The time course of induced TBA in the frontal region (the shaded region denotes the standard error of measurement [*SEM*]). At 600–750 ms after the probe item was presented, induced TBA was remarkably increased in the frontal region in patients with RLS

Figure 2 shows the group‐averaged time−frequency maps and topographical distributions of the induced TBA. The induced TBA in the frontal area was remarkably decreased, and the peak of the theta‐band power was delayed by ~50 ms in the RLS group compared with that of the control group. The main effects of group and memory load size were both significant. The induced TBA was marginally lower in the RLS group than in the control group regardless of memory load and location (*F*_4,88_ = 3.92, *p* = .06). As the memory load increased, the induced theta‐band power became higher irrespective of the group and region (*F*_2,44_ = 7.73, *p* < .01). The main effect of region was significant (*F*_2,44_ = 3.26, *p* = .05). The interaction between the group and memory load size was significant, whereas interactions between group and region and between memory load size and region were not significant (*F*_2,44_ = 5.86, *p* < .01; *F*_2,44_ = 0.04, *p* = .95; and *F*_4,88_ = 1.49, *p* = .22, respectively). The interaction among group, memory load size and region was not significant (*F*_4,88_ = 1.04, *p* = .38).

Post hoc analysis revealed that induced TBA for a higher memory load was significantly higher than that of a lower memory load in the RLS group (load size of 2 < load size of 4, *t*_11 _= −3.40, *p* < .01, Bonferroni corrected), whereas it was not significantly different with respect to memory load in the control group. For a memory load size of 3, the induced TBA was significantly lower in the RLS group than in the control group (*t*_22_ = 2.85, *p* < .01, Bonferroni corrected).

However, the difference in induced TBA between groups was marginally significant for a memory load size of 2 (*t*_22_ = 1.92, *p* = .06, Bonferroni corrected), and not significant for a memory load size of 4 (*t*_22_ = 0.12, *p* = .47, Bonferroni corrected).

### 3.4 Interregional TBPS

**FIGURE 3:**
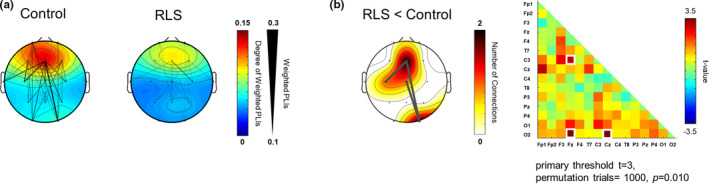
Interregional theta‐band phase synchrony (TBPS) during the memory retrieval phase. (a) Connection strength patterns between frontal and other regions at 600–700 ms in the control group and at 650–750 ms in the restless legs syndrome (RLS) group after the probe item were presented. To visualize the connectivity pattern more clearly, we only represented the connections whose weighted phase lag index (wPLI) was above 0.1. Lower connection strength centred on the frontal region was observed in patients with RLS. (b) Group differences in interregional phase synchrony were found in the theta band. The network‐based statistics (NBS) method was used to compare the topological properties of the brain network between the groups. Dark grey lines in the topography represent edges that were significantly decreased in the RLS group (left panel). The reduced network connections are evident predominantly in the frontal region. The elements of the adjacent matrix (right panel) are the *t*‐values for each connection, which were obtained from the group comparison. The white box in the matrix indicates the set of decreased functional connections in the RLS group

Figure 3(a) shows the spatial patterns of the interregional TBPS. Remarkable frontoparietal connectivity was observed 600–700 ms following the presentation of stimulation. The strength of the TBPS between the frontal and posterior regions was remarkably decreased in the RLS group compared with that of the control group. Group differences in interregional TBPS were adjusted by NBS (Figure 3b). A network of connections that showed a significant decrease in the RLS group was observed in the frontal region.

**FIGURE 4:**
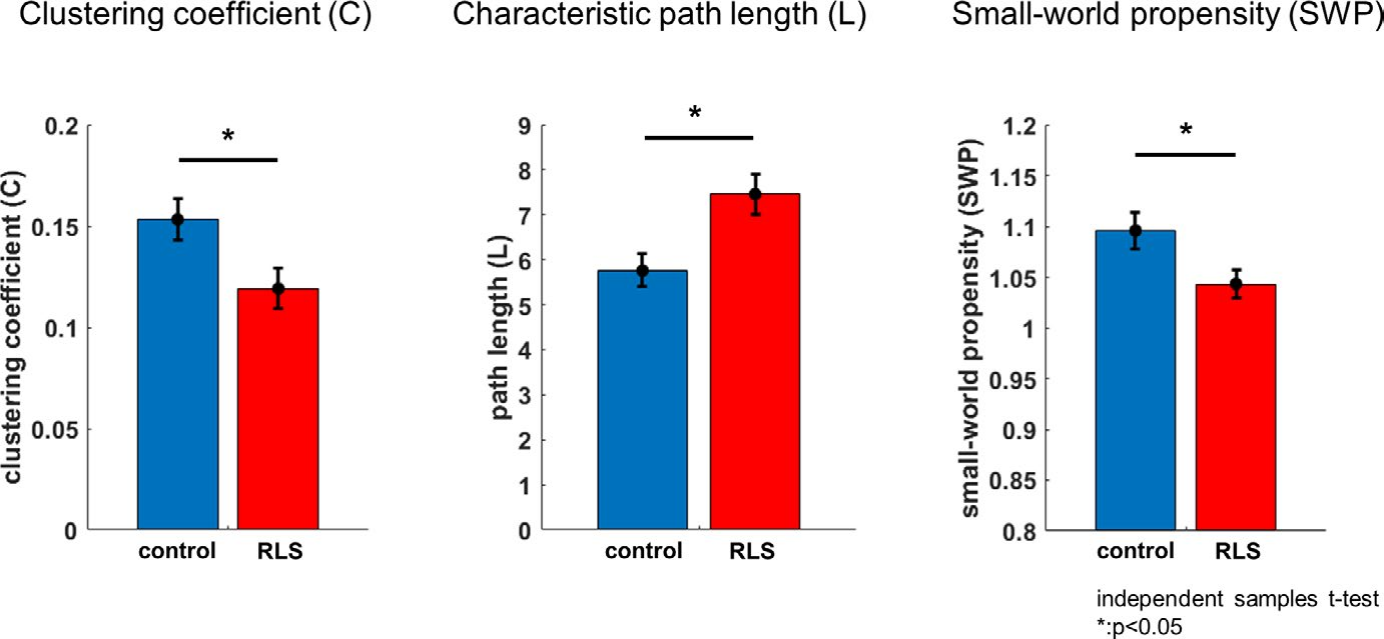
Graph theory analysis of theta‐band phase synchrony (TBPS). Graph theory indices, including the clustering coefficient (*C*), characteristic path length (*L*) and small‐world propensity (SWP), were analysed to evaluate network characteristics. These indices were calculated from the spatial pattern of TPBS at 600–700 ms in the control group and at 650–750 ms in the restless legs syndrome (RLS) group after the presentation of the probe item. Larger *L* and smaller *C* values were observed in the RLS groups (^*^ *p* < .05). Patients with RLS showed inefficiency in both local and global networks

Local connectedness, quantified by *C* (*t*_22_ = 2.31, *p* = .03, *d* = 0.94), and global connectedness, quantified by *L* (*t*_22 _= −2.79, *p* = .01, *d* = −1.14), were both significantly less efficient in the RLS group than in the control group (Figure 4). In addition, the overall effectiveness of the network for interregional communication, quantified by SWP, was significantly lower in the RLS group than in the control group (*t*_22_ = 2.26, *p* = .03, *d* = 0.92).

### 3.5 Correlation analysis

**FIGURE 5:**
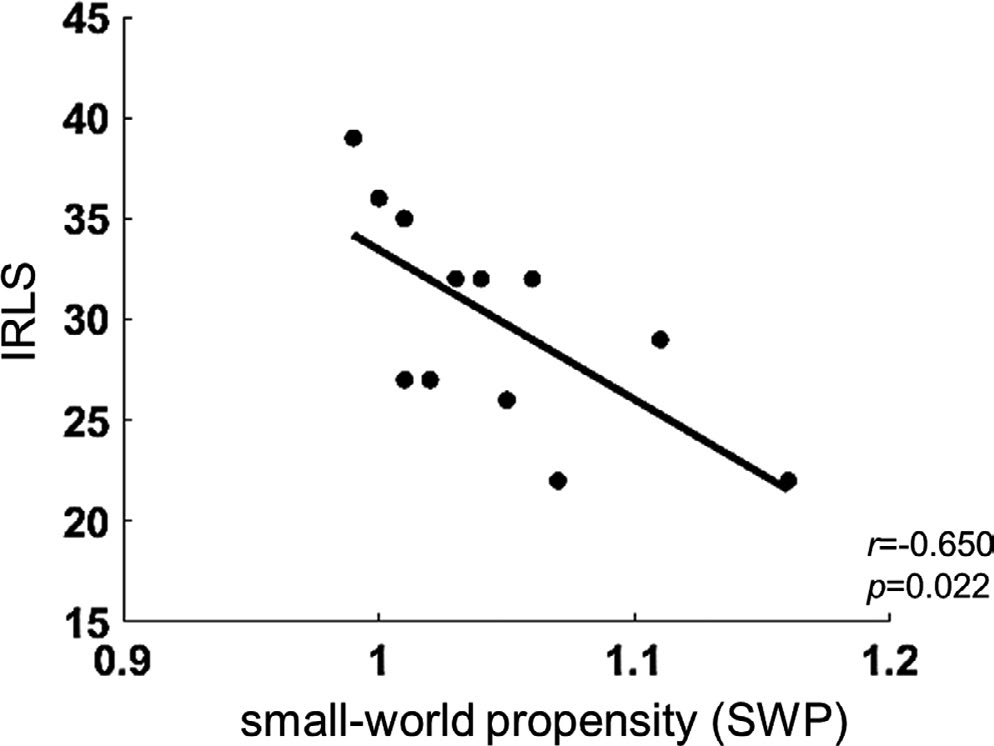
Correlation between small‐world propensity (SWP) and the International Restless Legs Syndrome Study Group (IRLSSG) rating scale (IRLS) score in the restless legs syndrome (RLS) group. SWP was negatively correlated with the IRLS score. An increase in network efficiency predicted reduced RLS severity

Small‐world propensity showed a significant negative correlation with IRLS (*r* = −.65, *p* = .02), whereas other clinical variables did not significantly correlate with SWP (Figure 5). TBA was not significantly correlated with any clinical variables (Table 3).

**TABLE 3:** Correlation analysis results of the RLS group

	Age	Duration	IRLS	ISI	PSQI	BDI‐II
Theta‐band power						
Spearman’s rho	0.004^a^	−0.218	−0.014	0.018	0.211	0.385
*p*‐value	.991	.497	.965	.957	.511	.216
SWP						
Spearman’s rho	−0.081	0.342	−0.650	−0.554	−0.236	−0.190
*p*‐value	.802	.278	.022	.062	.460	.555

BDI‐II, Beck Depression Inventory‐II; IRLS, International Restless Legs Syndrome Study Group Rating Scale; ISI, Insomnia Severity Index; PSQI, Pittsburgh Sleep Quality Index; RLS, restless legs syndrome; SWP, small‐world propensity.

^a^ Spearman’s correlation.

## 4 DISCUSSION

In the present study, we characterized abnormal local theta‐band neural synchrony and global theta‐band neural synchrony in patients with RLS while they performed a Sternberg WM task. We found a lower brain network efficiency during WM performance in patients with RLS, which was negatively correlated with RLS severity.

The TBA in the frontal area was increased in both groups during the memory retrieval phase of the Sternberg WM task. It is known that frontal TBA contributes to WM manipulation (Itthipuripat et al., 2013; Klimesch, 1999). In line with previous studies, the temporally enhanced TBA in the frontal area observed in our results may indicate memory manipulation processing involving encoded contents and evaluation of incoming visual information. It is remarkable that the patients with RLS showed a significantly smaller increment of TBA than the control subjects, which may signify impaired memory manipulation. RTs in patients with RLS were significantly longer than those in controls, whereas HRs were not different for all memory loads. Along with the reduced TBA, delayed RTs in patients with RLS may reflect deficits in the attention needed to manipulate WM.

Our results showed that the TBA changed with memory load, which may be related to task difficulty or the amount of encoded sensory information. A higher TBA for a high memory load was observed only in the RLS group. The enhanced frontal TBA associated with higher memory load may reflect an increase in mental effort and cortical resources due to task difficulty (Gevins et al., 1998; Jensen & Tesche, 2002). This indicates that patients with RLS require more attentional resources for a higher memory load, whereas sufficient cognitive capacity is available for any memory load in healthy controls due to the lower level of task difficulty.

Anterior‐posterior TBPS was also significantly increased in both groups. Increased theta‐band neural synchrony between the frontal and parietal regions is required for successful memory retrieval (Kim et al., 2012), which underlies top‐down control for memory retrieval and bottom‐up control for attentional capture by encoded memory (Fell & Axmacher, 2011; Sauseng et al., 2010). The anterior‐posterior TBPS was significantly reduced in the patients with RLS compared with the TBPS in control subjects. The fragmentation of interregional phase synchrony of theta‐band oscillations in patients with RLS may contribute to the deficit in information integration for the evaluation of probe stimuli and memory manipulation of encoded information. Consistent with the reduced functional connectivity strength, graph theory analysis revealed inefficient local and global connectedness in patients with RLS. Taken together, our results suggest that decreased WM performance, as demonstrated by longer RTs in patients with RLS, may result from multiple factors, including reduced attentional allocation, deficits in stimulus evaluation, and memory manipulation of encoded information. This finding is in agreement with our previous study, which showed that interregional neural synchrony in the gamma band during a visual oddball task was reduced in patients with RLS compared with that in healthy controls (Choi et al., 2012). To successfully retrieve WM, encoded memory traces should be reactivated by theta‐band synchronization between the hippocampus and mPFC (Backus et al., 2016). We previously observed mPFC abnormalities in patients with RLS during the utilization of memory content for target discrimination (Cha et al., 2017). Therefore, it is speculated that transient alterations in theta‐band neural synchrony when performing the Sternberg WM task may be associated with mPFC abnormalities in RLS.

In our study, only the IRLS score among clinical variables evaluated was significantly negatively correlated with network efficiency in patients with RLS. Our previous study revealed changes in the P300 amplitude in patients with RLS when they were performing a WM task (Kim et al., 2014). Sleep quality variables were not correlated with the P300 amplitude in patients with RLS. However, the P300 amplitude was negatively correlated with the duration of RLS history. We speculated that repetitive pain attacks characteristic of chronic pain disorders may cause plastic changes in the cerebral cortices. Unlike those results, our present findings have shown that brain network efficiency is correlated with RLS severity, which reflects the current symptoms that the patients suffer. The P300 amplitude, which is generated by local neural activity, may be reduced by plastic changes in the cerebral cortices arising from repetitive pain attacks. The brain network, which is generated by interregional neural synchrony, may be disrupted by the direct effect of RLS symptoms.

Brain network efficiency was marginally correlated with ISI scores, but was not correlated with PSQI scores. A less efficient brain network in patients with RLS may also be associated with the effect of sleep disturbance as well as RLS per se. This cannot be clearly explained, but the lack of correlation between network efficiency and PSQI scores may be caused by the small sample size.

Our findings should be interpreted with caution due to the small sample size, and further confirmation is required in studies with larger samples. The use of a small number of EEG electrodes is also a limitation of our study. A high‐density EEG system would be necessary to estimate brain structures precisely.

In conclusion, although WM performance revealed by neuropsychological tests remains uncertain, abnormal local theta‐band neural synchrony and global theta‐band neural synchrony may underlie the neurophysiological mechanism for WM dysfunction associated with RLS.

## CONFLICT OF INTEREST

No conflicts of interest declared.

## AUTHOR CONTRIBUTIONS

Kwang Su Cha, Jun‐Sang Sunwoo, Jung‐Ick Byun, Tae‐Joon Kim, Jung‐Won Shin, Kyung Hwan Kim and Ki‐Young Jung were responsible for study design and statistical analysis. Kwang Su Cha, Kyung Hwan Kim and Ki‐Young Jung contributed to write the manuscript. All authors have contributed to and have approved the final manuscript.

## Data Availability

The datasets generated and/or analyzed during the current study are available from the corresponding author upon reasonable request.
